# Teleconsultation in the Management of Elective Orthopedic and Spinal Conditions During the COVID-19 Pandemic: Prospective Cohort Study of Patient Experiences

**DOI:** 10.2196/28140

**Published:** 2021-06-15

**Authors:** Christina Melian, Christopher Frampton, Michael Charles Wyatt, David Kieser

**Affiliations:** 1 Renaissance School of Medicine at Stony Brook University Stony Brook, NY United States; 2 University of Otago Christchurch New Zealand; 3 Christchurch School of Medicine Christchurch New Zealand

**Keywords:** telemedicine, patient satisfaction, orthopedic surgery, telehealth, COVID-19, pandemic

## Abstract

**Background:**

The global adoption of teleconsultation has been expedited as a result of the COVID-19 pandemic. By allowing remote communication, teleconsultation may help limit the spread of the virus while maintaining the crucial patient-provider relationship.

**Objective:**

The aim of this study is to evaluate the value of teleconsultation compared to in-person visits in the management of elective orthopedic and spinal procedures.

**Methods:**

This was a prospective observational cohort study of 853 patients receiving orthopedic and spinal care at a private outpatient clinic in New Zealand. Patients were randomly divided into two groups: (1) patients receiving telephone consultation remotely, and (2) patients receiving in-person office consultations at the outpatient clinic. All patients received telephone consultations for 4 weeks during the mandated COVID-19 lockdown, followed by 4 weeks of telephone or in-person consultation. Patient preference, satisfaction, and duration of visit were recorded. Comparisons of patient preference between groups, visit type, sex, and location were performed using chi-square tests; similarly, satisfaction scores and visit durations were compared using a general linear model.

**Results:**

We report that 91% (353/388) of patients in the telephone group preferred teleconsultation over in-person office visits during the COVID-19 lockdown (*P*<.001). A combined-group analysis showed that 55.3% (446/807) of all patients preferred teleconsultation compared to 31.2% (252/807) who preferred in-person office visits (*P*<.001). Patients in the telephone group reported significantly higher satisfaction scores (mean 9.95, SD 0.04, 95% CI 9.87-10.03) compared to patients in the in-person group (mean 9.53, SE 0.04, 95% CI 9.45-9.62; *P*<.001). Additionally, in-person consultations were significantly longer in duration compared to telephone consultations, with a mean visit time of 6.70 (SE 0.18) minutes, 95% CI 6.32-7.02, compared to 5.10 (SE 0.17) minutes, 95% CI 4.73-5.42 (*P*<.001).

**Conclusions:**

Patients who use telephone consultations are more likely to prefer it over traditional, in-person visits in the future. This increased preference, coupled with higher patient satisfaction scores and shorter duration of visits, suggests that teleconsultation has a role in orthopedic surgery, which may even extend beyond the COVID-19 pandemic.

## Introduction

With the unexpected arrival of COVID-19, there has been a rapid uptake in the use of digital technology in health care, including orthopedic surgery [1,2]. By providing a secure platform for remote communication, teleconsultation permits patients and physicians to stay connected despite strict lockdown restrictions. Such technologies limit virus exposure and preserve limited hospital supplies, while maintaining continuity of care [3]. Teleconsultation can be conducted using either asynchronous or synchronous delivery methods [4]. Most teleconsultation delivery systems use an asynchronous “store and forward” approach in which patient information is electronically delivered to physicians, and responses can be generated later. Synchronous methods that allow for real-time delivery of health care, such as through videoconferencing or telephone interviews, are more favorable as they maintain the patient-provider relationship that may otherwise be compromised in a “store and forward” delivery system [5].

A strong patient-provider relationship enhances patient satisfaction, compliance, and overall health outcomes [6]. In a recent systematic review and meta-analysis, we demonstrated that teleconsultation was equivocal to traditional face-to-face office visits in regard to patient and physician preference and satisfaction [7]. In fact, we found that patients who used teleconsultation were roughly 1.5 times more likely to prefer it for subsequent appointments over traditional office visits, indicating a potential role for such technologies beyond COVID-19.

The purpose of this study is to evaluate patient perceptions of telephone consultations compared to traditional, in-person consultations in the management of elective orthopedic and spinal procedures. We assess patient preference, satisfaction, and duration of consultation, hypothesizing that teleconsultation is comparable to in-person consultation in these regards. Consistent with the literature, we also hypothesize that first-hand exposure to teleconsultation will positively influence a patient’s preference for its use in the future [7].

## Methods

### Study Protocol

A four-level national lockdown alert system was introduced in New Zealand for the COVID-19 outbreak. From a medical perspective, level 1 permitted normal interactions and consultations. In contrast, level 4 meant no in-person contact for elective care. The level 4 New Zealand national lockdown for COVID-19 was between March 25 and April 27, 2020. Following this, New Zealand moved down alert levels and entered level 1 on June 8, 2020. New Zealand remained at alert level 1 until August 12, 2020.

This was a prospective observational cohort study of 853 patients (10-94 years old) evaluated at a private outpatient clinic in New Zealand for orthopedic and spinal procedures. The first cohort included 364 patients who had teleconsultations during the four weeks of level 4 lockdown (March 25 to April 27, 2020). The comparator group comprised 487 patients who had in-person consultations during the first four weeks of level 1 (June 8 to July 6, 2020). Consultations were conducted by two orthopedic surgeons (DK and MW). All patients were reviewed within five days of referral for new patients and a designated two-week or six-week appointment postoperatively for postoperative patients. Follow-up patients were reviewed either at six weeks or after an investigation was obtained. No change in this schedule occurred between the two groups.

Consultation durations were recorded as phone call duration or time from entry to exit in the consultation room. Patients were contacted, either by telephone or email, by the physician assistant within two weeks of their consultation to rate their satisfaction and preference for either teleconsultation or in-person visit. Evaluations were rated on a scale of 0-10, with 0 being the worst experience and 10 being the best experience possible. Preference was obtained via a 3-item questionnaire (phone, in-person, none) at final follow-up. All patients in the teleconsultation group received in-person follow-up within three months of their teleconsultation to ensure that the diagnosis and management was deemed appropriate by the treating clinician.

Informed patient consent was obtained from each patient. Ethical approval was sought but deemed unnecessary as this was part of a clinic audit.

### Outcomes

The primary outcome measures in this study were patient preference, satisfaction, and duration of consultation.

### Statistical Analysis

The percentages of participant preference for in-person, phone, or no preference were compared between in-person and phone consults using chi-square tests. Location was categorized into city dwellers (>30 minutes of travel time) and rural dwellers (<30 minutes of travel time). Similarly, comparisons of preferences between sex, consultation type, and location were compared using chi-square tests. The scores assigned to each consultation (0-10) and the durations of the consultations were compared between in-person and telephone consults, sex, consultation type, and location using a general linear model incorporating all factors in a single model for each outcome measure. Least square means derived from these analyses with 95% CIs are used to summarize these analyses. A two-tailed *P* value <.05 is taken to indicate statistical significance and all analyses were undertaken using SPSS (version 25.0; IBM Corp).

## Results

### Overview

In total, 14 of 364 total patients (3.8%) in the teleconsultation group did not attend their telephone appointment during the four-week level 4 lockdown; these same patients did not respond to the follow-up questionnaire assessing patient preference and satisfaction. Overall, 18 of 487 total patients in the in-person group (3.5%) did not attend their office visit during the first four weeks of the level 1 lockdown, and 32 patients (6.6%) did not respond to the follow-up questionnaire. Therefore, patient preference and satisfaction were reported by 807 patients, with the exception of preference by location; 32 patients reported mixed abode and were excluded from this measure.

There were two cases for which the surgeons felt the telephone consult was inappropriate: two initial consultations of coccydynia, due to the sensitive location of the pain and uncomfortable conversation that would best be delivered in-person. In addition, during the teleconsultations, two patients were incorrectly diagnosed as L5 radiculopathy, which when reviewed in person were clearly greater trochanteric pain syndrome, both cases of which resolved with a trochanteric bursal steroid injection. In addition, two patients in the teleconsultation group showed up in-person and were therefore counted as in-person visits.

### Patient Preference

**Figure 1:**
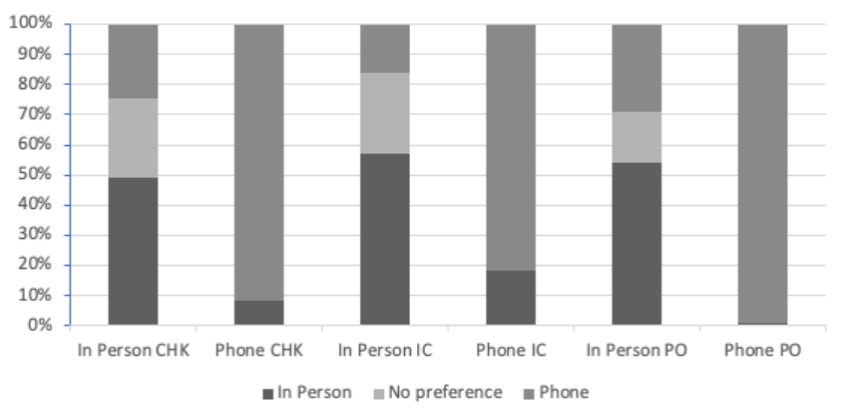
Preference for consultation by visit type. CHK: checkup; IC: initial consult; PO: postoperative.

Table 1 shows the overall patient preference for consultation in both the in-person and telephone group during the COVID-19 lockdown. The vast majority of patients in the telephone group (353/388, 91%) preferred teleconsultation over in-person office visits compared to 51.8% (217/419) of patients in the in-person group who preferred in-person office visits. When looking at the combined groups, 55.3% (446/807) of patients preferred teleconsultation over in-person visits compared to 31.2% (252/807) who preferred in-person office visits (*P*<.001). Teleconsultation was preferred over in-person office visits by 87.5% (126/144) of those evaluated postoperatively, as well as 48.5% (200/412) and 47.8% (120/251) of patients receiving checkups and initial consults, respectively (Figure 1). In contrast, those who preferred in-person office visits were mostly evaluated for initial consults (96/251, 38.2%), followed by checkups (142/412, 34.5%) and postoperative visits (14/144, 9.7%). No preference for either teleconsultation or in-person visits was reported in 13.5% (109/807) of total patients.

**Figure 2:**
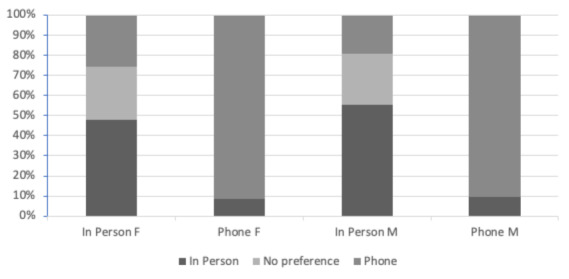
Preference for consultation by sex. M: male; F: female.

When analyzed by sex, both males and females reported a stronger preference for teleconsultation over in-person office visits (Figure 2). Out of all the patients evaluated, 54.5% (234/429) of males and 56.1% (212/378) of females chose teleconsultation over in-person visits for future visits (Table S1 in Multimedia Appendix 1; *P*<.001). In fact, roughly 90% of both males and females in the telephone group indicated that they would prefer teleconsultation for subsequent visits.

**Table 1:** Patient preference for consultation according to visit type.

Visit type			Consult preference							*P* value
			In person		No preference		Phone			
**Checkup (n=412), n (%)**								<.001		
	In person	130 (49.1)		70 (26.4)		65 (24.5)				
	Phone	12 (8.2)		0 (0)		135 (91.8)				
	Total	142 (34.5)		70 (17)		200 (48.5)				
**Initial consult (n=251), n (%)**								<.001		
	In person	74 (56.9)		35 (26.9)		21 (16.2)				
	Phone	22 (18.2)		0 (0)		99 (81.8)				
	Total	96 (38.2)		35 (13.9)		120 (47.8)				
**Postoperative (n=144), n (%)**								<.001		
	In person	13 (54.2)		4 (16.7)		7 (29.2)				
	Phone	1 (0.8)		0 (0)		119 (99.2)				
	Total	14 (9.7)		4 (2.8)		126 (87.5)				
**Total (n=807), n (%)**								<.001		
	In person	217 (51.8)		109 (26)		93 (22.2)				
	Phone	35 (9)		0 (0)		353 (91)				
	Total	252 (31.2)		109 (13.5)		446 (55.3)				

**Figure 3:**
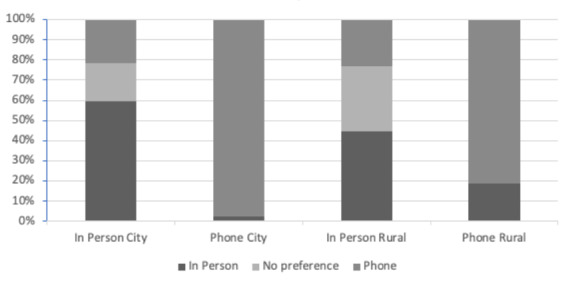
Preference for consultation by location. City: <30 minutes of travel; rural: >30 minutes of travel.

When analyzed by location, teleconsultation was preferred over in-person office visits by both city and rural community dwellers (Figure 3). Out of all the patients evaluated, 58.9% (234/397) of patients living in the city preferred teleconsultation compared to 47.6% (180/378) of patients living in a rural community (Table S2 in Multimedia Appendix 1; *P*<.001). A striking 97.4% (191/196) of city dwellers in the telephone group preferred teleconsultation over in-person office visits compared to 81.3% (130/160) of patients living in a rural community.

### Patient Satisfaction

Overall, there was a significantly higher satisfaction rating among the telephone group (mean 9.95, SE 0.04, 95% CI 9.87-10.03; *P*<.001) compared to the in-person group (mean 9.53, SD 0.04, 95% CI 9.45-9.62; *P*<.001; Table 2). A significant difference between the different types of visits was observed, with checkup patients reporting the highest satisfaction (mean 9.82, SE 0.04, 95% CI 9.74-9.89), followed by postoperative (mean 9.78, SE 0.07, 95% CI 9.65-9.92) and initial consultations (mean 9.62, SE 0.05, 95% CI 9.53-9.72; *P*=.006). No significant difference was detected between groups in regard to sex or location.

**Table 2:** Patient satisfaction (on a scale from 0-10, with 10 being the most satisfied) according to sex, location, consultation, and visit type.

Variables		Mean (SE)	95% CI	
			Lower bound	Upper bound
**Sex**				
	Female	9.73 (0.04)	9.64	9.81
	Male	9.75 (0.04)	9.68	9.83
**Location**				
	City (<30 minutes of travel time)	9.71 (0.04)	9.64	9.79
	Rural (>30 minutes of travel time)	9.77 (0.04)	9.68	9.85
**Consultation**				
	In person	9.53 (0.04)	9.45	9.62
	Phone	9.95 (0.04)	9.87	10.03
**Type**				
	Checkup	9.82 (0.04)	9.74	9.89
	Initial consult	9.62 (0.05)	9.53	9.72
	Postoperative	9.78 (0.07)	9.65	9.92

### Duration of Consultation

In-person consultations were significantly longer in duration compared to telephone consultations, with a mean visit time of 6.70 (SE 0.18) minutes, 95% CI 6.32-7.02, and 5.10 (SE 0.17) minutes, 95% CI 4.73-5.42, respectively (*P*<.001). Initial consultations took the longest to conduct (8.50 minutes, SE 0.20 minutes, 95% CI 8.067-8.87), followed by checkup (5.0 minutes, SE 0.16 minutes, 95% CI 4.73-5.37) and postoperative visits (4.10 minutes, SE 0.29 minutes, 95% CI 3.54-4.67; *P*<.001). No significant difference in consultation duration was observed in regard to sex or location.

## Discussion

We report an increased preference for teleconsultation, greater patient satisfaction, and shorter duration of visits in patients who had telephone consultations during the COVID-19 lockdown. Studies have shown that a strong patient-physician relationship is correlated with greater medical adherence and positive health outcomes [6]. However, the abrupt onset of the COVID-19 pandemic has threatened this fundamental relationship by limiting in-person consultations and impeding communication between patients and physicians. Teleconsultation offers a potential solution by providing a platform through which patients and physicians can establish and maintain communication to better manage elective orthopedic and spinal conditions [2,8]. Despite the advantages of teleconsultation, one of the biggest threats to its implementation is patient satisfaction and willingness to adopt such new technologies [9,10]. Previous studies suggest that patient preference and satisfaction are key indicators of how effective teleconsultation modalities will be in clinical practice [9].

In this study, we compare the patient perception of teleconsultation with that of traditional, in-person consultations in the management of elective orthopedic and spinal procedures during the COVID-19 lockdown. We found that patients receiving telephone consultation had a significantly higher preference for teleconsultation than those receiving in-person visits, regardless of the type of visit (ie, checkup, initial, postoperative). Similarly, patients receiving in-person consultation had a higher preference for in-person consultation, suggesting that familiarity and convenience may play a role in patient preference for consultation type [11]. A closer look at the data reveals the greatest preference for teleconsultation is among patients presenting postoperatively, followed by those undergoing checkup visits and initial consultations, respectively. This is consistent with the literature showing that patients are more likely to prefer teleconsultation for follow-up appointments, as opposed to primary encounters, given the nature of the visits [12]. Initial consultations tend to be more thorough, with the focus being on building rapport between patient and physician [13]. As a result, patients may be more resistant to disclose personal information via telephone if a strong patient-physician relationship has not already been established. This is in contrast to postoperative or checkup visits in which a strong relationship has most likely been achieved at prior visits, making teleconsultation a suitable method of care. Interestingly, we found that patients living in the city (<30 minutes of travel time) were more likely to prefer teleconsultation compared to patients living in rural communities (>30 minutes of travel time). One might assume that patients living in rural communities would have a stronger preference for teleconsultation given the health disparities typically seen in rural communities [14]. We hypothesize that this is due to the busier lifestyles and greater time demands experienced by city dwellers, thus making remote consultations more desirable.

In terms of patient satisfaction, statistically higher levels were achieved in the telephone consultation group compared to the in-person group, across all visit types. Such findings may be indirectly linked to the significantly shorter duration of visits observed among the teleconsultation group. Not surprisingly, initial consultations took the longest to conduct, followed by checkups and postoperative visits, respectively. Although not assessed in this study, the higher patient satisfaction observed in the teleconsultation group may also be attributed to a reduction in travel time [15], cost reduction [16], and improved access to care [16].

While these results show strong evidence in favor of teleconsultation, this study has a few limitations. First, telephone consultations were performed during a mandated lockdown when people were required to stay at home, without the option for in-person reviews. Therefore, we must consider the impact of the COVID-19 pandemic on patients’ preference for teleconsultation. It is possible that patients would have preferred in-person consultation if the pandemic was not a threat. For this reason, a re-review would be prudent outside of the COVID-19 pandemic to reinforce the results of this study. Second, this study was conducted at a single outpatient clinic in New Zealand. Additionally, patient preference and satisfaction ratings were assessed through self-reported measures, with 78 patients lost to follow-up.

Despite such limitations, our results suggest that teleconsultation may have real therapeutic value in the management of orthopedic and spinal conditions. From a patient perspective, teleconsultation does not appear to be inferior to traditional, in-person office visits in terms of preference and overall satisfaction. Although this study was conducted during the mandated COVID-19 lockdown, it should be noted that teleconsultation has been on the rise across health care fields internationally for the past decade. Therefore, our findings further support the use of teleconsultation, even beyond the COVID-19 pandemic.

## Appendix

Multimedia Appendix 1 Supplementary data.
