# Supplementary material for: Teleconsultation in the Management of Elective Orthopedic and Spinal Conditions During the COVID-19 Pandemic: Prospective Cohort Study of Patient Experiences
Source: JMIR Form Res. 2021 Jun 15;5(6):e28140. doi: 10.2196/28140 (PMC8208469; doi:10.2196/28140)
Supplement: Multimedia Appendix 1 [file formative_v5i6e28140_app1.docx]

## Supplementary Data

**Table S1.** Patient preference for consultation according to sex; F: female, M: male, CHK: check-up, IC: initial consult, PO: post-op

| Sex Visit Type | | Consult Preference | | |  |
| --- | --- | --- | --- | --- | --- |
|  |  | In Person (%) | No preference (%) | Phone (%) | P-value |
| **Female**  (n=378) | In Person | 97 (47.8) | 54 (26.6) | 52 (25.6) | 0.000 |
|  | Phone | 15 (8.6) | 0 | 160 (91.4) |  |
|  | Total | 112 (29.6) | 54 (14.3) | 212 (56.1) |  |
| **Male**  (n=429) | In Person | 120 (55.6) | 55 (25.5) | 41 (19) | 0.000 |
|  | Phone | 20 (9.4) | 0 | 193 (90.6) |  |
|  | Total | 140 (32.6) | 55 (12.8) | 234 (54.5) |  |

**Table S2.** Patient preference for consultation according to location; City: < 30 minutes travel, Rural: >30 minutes travel, CHK: check-up, IC: initial consult, PO: post-op

| Location Visit Type | | Consult Preference | | |  |
| --- | --- | --- | --- | --- | --- |
|  |  | In Person (%) | No preference (%) | Phone (%) | P-value |
| **City**  (n=397) | In Person | 120 (59.7) | 38 (18.9) | 43 (21.4) | 0.000 |
|  | Phone | 5 (2.6) | 0 | 191 (97.4) |  |
|  | Total | 125 (31.5) | 38 (9.6) | 234 (58.9) |  |
| **Rural**  (n=378) | In Person | 97 (44.5) | 71 (32.6) | 50 (22.9) | 0.000 |
|  | Phone | 30 (18.8) | 0 | 130 (81.3) |  |
|  | Total | 127 (33.6) | 71 (18.8) | 180 (47.6) |  |
